# Persistent Non-pharmacological Pain Management and Brain-Predicted Age Differences in Middle-Aged and Older Adults With Chronic Knee Pain

**DOI:** 10.3389/fpain.2022.868546

**Published:** 2022-07-12

**Authors:** Alisa J. Johnson, James Cole, Roger B. Fillingim, Yenisel Cruz-Almeida

**Affiliations:** ^1^Pain Research and Intervention Center of Excellence, University of Florida, Gainesville, FL, United States; ^2^Department of Community Dentistry and Behavioral Science, College of Dentistry, University of Florida, Gainesville, FL, United States; ^3^Centre for Medical Image Computing, Department of Computer Science, University College London, London, United Kingdom; ^4^Dementia Research Centre, Institute of Neurology, University College London, London, United Kingdom

**Keywords:** chronic musculoskeletal pain, non-pharmacological, brain age, clinical pain, knee osteoarthritis

## Abstract

Chronic pain has been associated with changes in pain-related brain structure and function, including advanced brain aging. Non-pharmacological pain management is central to effective pain management. However, it is currently unknown how use of non-pharmacological pain management is associated with pain-related brain changes. The objective of the current study was to examine the association between brain-predicted age difference and use of non-pharmacological pain management (NPM) in a sample of middle-aged and older adults with and without chronic knee pain across two time points. One-hundred and 12 adults (mean age = 57.9 ± 8.2 years) completed sociodemographic measures, clinical pain measures, structural T1-weighted brain magnetic resonance imaging, and self-reported non-pharmacological pain management. Using a validated approach, we estimated a brain-predicted age difference (brain-PAD) biomarker, calculated as brain-predicted age minus chronological age, and the change in brain-PAD across 2 years. Repeated measures analysis of covariance was conducted to determine associations of non-pharmacological pain management and brain-PAD, adjusting for age, sex, study site, and clinical pain. There was a significant time^*^pain/NPM interaction effect in brain-PAD (*p* < 0.05). Tests of simple main effects indicated that those persistently using NPM had a “younger” brain-PAD over time, suggesting a potential protective factor in persistent NPM use. Future studies are warranted to determine the influence of NPM in brain aging and pain-related neurological changes.

## Introduction

Chronic musculoskeletal (MSK) pain has been associated with changes in brain structure (1, 2) and function (3–5), including indications of advanced age-related brain atrophy (6–8). While aging is typically associated with neurological changes (9) even in healthy adults (10), there is wide variation in brain aging, which may place some individuals at a greater risk for functional loss and worse health outcomes. In our previous work, we employed an established brain aging biomarker, using the “brain-predicted age difference” paradigm (11), and found that older adults with various types of chronic pain had an ‘older' appearing brain age compared to those with no chronic pain reports in the past 3 months (6), suggesting an additive effect of chronic pain on age-related brain changes. This is in contrast to findings by Soros et al. (12) suggesting chronic pain was not associated with advanced brain-aging. One explanation for these different findings may be in the pain-seeking behavior of the study samples. In our study, we included older healthy adults from the community reporting mainly non-pharmacological pain management (NPM) strategies, while the participants in the Soros and colleagues study were recruited from a pain clinic and did not report on the use of NPM. While chronological aging is accompanied by gray matter decline, which may be exacerbated by living with chronic pain, use of NPM may provide protection against age and/or pain-related brain atrophy (13, 14). However, these relationships are not well-understood, particularly among commonly used therapies in persons with knee OA (e.g., heat, ice, ointments, therapeutic massage).

The purpose of the current study was to examine the association between a marker of brain aging [i.e., brain-predicted age difference (brain-PAD)] and use of common NPM strategies in a sample of middle-aged and older adults with and without chronic knee-OA related pain. We examined the association of persistent NPM use over time (i.e., NPM use reported at both baseline and two-year follow-up) to determine associations with changes in brain aging. In the current study, NPM was operationalized as non-drug interventions participants self-reported using to manage knee pain. Four specific physically oriented interventions were queried: heat, ice, ointments, and massage therapy. Based on our prior work and that of others, we hypothesized that NPM would be associated with a significantly “younger” brain age at baseline and less brain aging over a two-year time period.

## Materials and Methods

### Participants

Participants were recruited as part of a larger, prospective, multisite study, conducted at the University of Florida (UF) and the University of Alabama, Birmingham (UAB). The study was approved by the UF and UAB Institutional Review Boards and all participants provided written informed consent. Power calculations were conducted for the larger parent study a priori. For the current analysis, we were powered (1-β = 0.99, two-sided α = 0.05) to detect a medium effect size ($n_{p}^{2}=$ 0.06), for time^*^pain/NPM group interaction on brain-PAD. The current study included English speaking, non-Hispanic “Black/African American” (NHB; 46.9%) and “White/Caucasian” (NHW; 53.1%) adults between the ages of 45–85 years, with (*n* = 94) and without (*n* = 19) chronic knee pain. Participants were recruited using fliers, radio and print ads, word of mouth, and clinical referrals and compensated for their participation. Full inclusion/exclusion criteria have been previously published (15). Briefly, participants were excluded if they reported: (1) prosthetic knee or significant surgery to the most painful knee, (2) uncontrolled hypertension, (3) cardiovascular disease, (4) serious psychiatric disorder, (5) neurological diseases, (6) peripheral neuropathy, (7) systemic rheumatic diseases other than osteoarthritis, (8) pregnant or nursing, (9) daily opioid use, and (10) significantly greater body pain in a site other than the knee. Participants were classified for analysis based on self-reported chronic knee pain (i.e., pain experienced over the past 6 months), and use of non-pharmacological pain management (NPM) at two separate time points separated by 2 years, resulting in three groups: (1) pain free, (2) chronic knee pain and persistent NPM use, (3) chronic knee pain and not persistent NPM use.

### Procedures

The study consisted of two time points (i.e., baseline and two-year follow-up), separated by approximately 2 years. Individuals completed an initial phone screening (e.g., age, sex, and ethnicity/race, chronic knee pain) at baseline and were scheduled for a health assessment session (HAS), and a magnetic resonance imaging session (MRI), scheduled approximately 2 weeks apart. The HAS and MRI sessions were repeated at two-year follow-up. The HAS consisted of written informed consent, sociodemographic (e.g., age, ethnicity/race, sex, education level, income) and general health and pain history questionnaires (e.g., current health conditions, pain sites). Clinical pain measures (i.e., Graded Chronic Pain Scale), were completed within 24 h prior to the MRI session. Both time points consisted of the same procedures.

#### Magnetic Resonance Imaging (MRI) Session

Neuroimaging was conducted at the University of Florida's McKnight Brain Institute using a 3T Phillips Achieva (Best, Netherlands) scanner with a 32-channel head coil, and a 3T Phillips Achieva (Best, Netherlands) at the University of Alabama, Birmingham using an 8-channel head coil. T1-weighted (T1w) anatomical scans were conducted using a high-resolution 3D MP-RAGE sequence collected using the following parameters: repetition time = 7.0 ms; echo time = 3.2 ms, 176 slices in a sagittal orientation, 8° flip angle, resolution = 1 mm^3^, FOV:240 x 240 x 176. Cushions were placed inside the head coil and instructions given to participants to minimize head movement during scanning.

### Measures

#### Brain-Predicted age Difference (Brain-PAD)

We utilized the established brain age biomarker developed by Cole and Franke (11), which uses supervised machine learning to accurately predict chronological age from multivariate structural neuroimaging data (11). The brainageR model v2.1 generates brain-predicted age values from raw T1-wieghted MRI neuroimaging scans, using SPM12 for segmentation and normalization. Normalized images are then loaded into R using the *RNFti* package, vectorised and gray matter, white matter, and CSF vectors masked and combined. Principal components analysis was applied (R prcomp), and the top 80% of variance retained, which resulted in 435 principal components that were then used to predict an age value with the trained model with R's *kernlab*, as described here https://github.com/james-cole/brainageR. The training cohort consisted of 3,377 healthy adults (age range = 18–92 years, mean age = 40.6 ± 21.4 years), free of neurological and psychiatric disorders, with no history of head trauma or other major medical conditions, obtained from seven public datasets (16), including the Australian Imaging, Biomarker & Lifestyle Flagship Study of Aging (AIBL), Dallas Lifespan Brain Study (DLBS), Brain Genome Superstruct Project (GSP), IXI, Nathan Kline Institute Rocklands Sample Enhanced, Open Access Series of Imaging Studies-1 (OASIS-1), and the Southwest University Adult Lifespan Dataset (SALD). The independent test dataset consisted of 857 healthy adults, ranging in age from 18–90 years (mean age = 40.1 ± 21.8 years). Ethical approval for each initial study and data sharing was verified for each data repository. Tests of model accuracy using held-out test data (with random assignment to training and test groups) were high, with a mean absolute error of 3.93 years and a correlation of r = 0.97, R^2^ = 0.95, between chronological age and “brain-predicted age”. We used the regression model trained on the full independent dataset (*n* = 3,377) to generate brain-predicted aged values for the participants in the current study (*n* = 112). As with our previous study (6), we subtracted each participant's chronological age from their brain-predicted age value to calculate a brain-predicted age difference (brain-PAD) score for each study time point (i.e., baseline and follow-up). Greater values indicate an “older” brain age.

#### Chronic Pain

The Graded Chronic Pain Scale (GCPS) (17) consists of three items that ask individuals to rate their current pain, and worst and average pain over the past 6 months, on a 0 (no pain) to 10 (worst pain imaginable) numerical rating scale (NRS). Items were averaged and multiplied by 10 to produce a characteristic pain intensity score ranging from 0–100, with higher scores indicating greater pain. Three questions asked participants to rate how much pain has interfered with daily activities, recreational, social and family activities, and ability to work over the past 6 months. Items were scored on a 0 (no interference) to 10 (unable to carry out activity) NRS, averaged and multiplied by 10 to produce a global pain-related disability score. Higher scores indicate greater pain-related disability. One item asks participants to indicate the number of days in the past 6 months that they have been kept from their usual activities due to pain. Pain-related disability points are calculated from the scale score and the total number of days reported as follows: (1) the averaged ratings (i.e., 0–29 = 0 points; 30–49 = 1 point; 50–69 = 2 points; ≥ 70 = 3 points), and (2) total number of disability days (i.e., 0–6 days = 0 points; 7–14 days = 1 point; 15–30 days = 2 points; 31 days or more = 3 points). Pain grades were computed as the sum of disability points and the presence/absence of chronic pain: Grade 0 = no reported pain intensity; Grade 1 = low pain intensity (i.e., <50) and low disability (i.e., <3 disability points); Grade 2 = high intensity pain (i.e., ≥ 50) and low disability; Grade 3 = high disability-moderately limiting (i.e., 3–4 disability points), regardless of pain intensity; Grade 4 = high disability-severely limiting (i.e., 5–6 disability points), regardless of pain intensity (17).

Participants also self-reported the length of time they had been experiencing pain in their knee (i.e., Pain Duration) using a Likert-type scale with the following response choices: 1 = <6 months, 2 = 6 months to 1 year, 3 = 1 to 3 years, 4 = 3 to 5 years, 5 = > 5 years.

Non-pharmacological Pain Management (NPM). Participants were asked to report if they used any of the following non-pharmacological modalities: (1) ice, (2) heat, (3) ointments, (4) massage, and/or (5) other, to manage their pain. If “other” was selected, participants were asked to write in the non-pharmacological treatment (NPM) they used. NPM use at baseline and two-year follow-up was coded as 0 (“no use”), and 1 (“use”). Among those reporting chronic pain at both time points, we identified individuals endorsing NPM at both baseline and two-year follow-up (persistent users), and those not reporting NPM at both time points (not persistent users).

### Statistical Analysis

Data analysis was performed in SPSS vs.27.0 (IBM). Data were checked for anomalies prior to analysis. Mean and standard deviation were used to summarize continuous data. Frequency statistics are reported for categorical data. Assumptions for each statistical test were tested. One-way analysis of variance (ANOVA) and Chi-square were used to compare groups on continuous/discrete ordinal and categorical data, ,respectively, for sociodemographic (i.e., age, sex, race, study site, education, and income), and clinical (i.e., knee pain duration) characteristics. Individuals were classified based on the presence/absence of pain (i.e., pain free and chronic knee pain), and if they persistently/not persistently reported using NPM (in those with chronic knee pain). We examined pain/NPM group differences in brain-PAD using a 2 (time) x 3 (group) repeated measures analysis of covariance (RM-ANCOVA), with time (i.e., baseline and two-year follow-up) as the within-subjects factor, and pain/NPM use group (i.e., pain free, chronic pain and persistent NPM use, and chronic pain and non-persistent NPM use) as the between subjects factor, adjusted for age, sex, study site, and pain duration reported at two-year follow-up. In secondary analyses, RM-ANCOVA was used to assess NPM group changes in GCPS pain intensity and pain-related disability in those with chronic pain, adjusting for age, study site, and sex. Tests of simple main effects with Bonferroni correction for multiple comparisons were conducted. A probability of < 0.05 was considered statistically significant. Greenhouse-Geisser correction was reported when sphericity assumption was violated. Partial eta squared was used to assess the magnitude of the effect with 0.01, 0.06, and 0.14 representing small, medium, and large effect sizes, respectively (18).

## Results

### Sample Demographics

The present study sample is comprised of 113 middle-aged and older adults (mean age = 57.9 ± 8.2 years) with and without chronic knee pain who completed neuroimaging and measures of interest at both time points. 15 individuals were excluded from analysis due to a change in pain status (e.g., pain free to pain) over the two-year study period. At baseline, the majority of those with chronic pain (61.1%) reported using at least one type of non-pharmacological pain management (NPM) strategy. Sample demographics and clinical pain characteristics at baseline are presented in Table 1. There were no significant differences between groups for age, education, or income (*p*'s > 0.05). However, a greater proportion of females with chronic pain reported persistent NPM use than males [χ^2^(2) = 9.65, *p* = 0.008]. The type of NPM strategies employed by persistent and non-persistent NPM users is presented in Table 2.

**Table 1:** Sample demographic and clinical characteristics at baseline.

**Variable, M (SD) or *N* (%)**	**Pain free** **(*n* = 19)**	**Chronic pain persistent NPM** **(*n* = 60)**	**Chronic pain** **not persistent NPM (*n* = 34)**	***P***
Chronological age	58.6 (9.3)	58.1 (7.7)	57.1 (8.5)	0.778
Brain-predicted age	54.7 (12.8)	56.0 (9.6)	55.2 (10.9)	0.840
Sex				**0.008**
Male	6 (31.6)	13 (21.7)	18 (51.5)	
Female	13 (68.4)	47 (78.3)	16 (48.5)	
Ethnicity/race				**0.039**
NHB	9 (47.4)	34 (56.7)	10 (29.4)	
NHW	10 (52.6)	26 (43.3)	24 (70.6)	
Education				0.195
< High school	0 (0)	6 (10.0)	1 (2.9)	
High school	4 (21.1)	23 (38.3)	10 (29.4)	
Two-year college	7 (36.8)	9 (15.0)	4 (11.8)	
Four-year college	4 (21.1)	15 (25.0)	11 (32.4)	
Master's	3 (15.8)	5 (8.3)	4 (11.8)	
Doctoral	1 (5.3)	2 (3.3)	4 (11.8)	
Income				0.248
< $20k	4 (21.1)	18 (31.6)	10 (29.4)	
$20–$49,999k	2 (10.5)	17 (29.8)	5 (15.1)	
$50–$79,999k	7 (36.8)	11 (19.3)	7 (22.1)	
$>80k	6 (31.6)	11 (19.3)	11 (33.4)	
Study site				0.784
UF	11 (57.9)	40 (66.7)	22 (52.9)	
UAB	8 (42.1)	20 (33.3)	12 (47.1)	
Knee pain duration at follow-Up*				**0.001**
<6 mth	1 (100.0)	0 (0)	3 (8.8)	
6 mth–1 year	0 (0)	1 (1.7)	0 (0)	
1–3 years	0 (0)	7 (11.7)	3 (8.8)	
3–5 years	0 (0)	12 (20.0)	6 (17.6)	
> 5 years	0 (0)	35 (58.3)	18 (52.9)	

* *Denotes missing data. NHB, non-Hispanic Black; NHW, non-Hispanic White; UF, University of Florida; UAB, University of Alabama, Birmingham; mth, months. Bolded values indicate statistical significance at α < 0.05*.

**Table 2:** Type of non-pharmacological pain management reported at each time point by pain/NPM group.

**Intervention, *N* (%)**	**Chronic pain/ Persistent NPM**		**Chronic pain/ Non-persistent NPM**	
	**(*****n*** **=** **60)**		**(*****n*** **=** **34)**	
	**Baseline**	**2-yr**	**Baseline**	**2-yr**
Ice	29 (48.3)	26 (43.3)	4 (12.1)	4 (12.1)
Heat	26 (43.3)	22 (36.7)	2 (6.1)	8 (23.5)
Ointments	27 (45.0)	38 (63.3)	2 (6.1)	2 (5.9)
Massage	29 (48.3)	23 (38.3)	3 (9.1)	6 (17.6)
Other*	12 (20.0)	17 (30.0)	2 (5.9)	2 (5.9)
Chiropractic	1 (1.7)	2 (3.3)	0 (0)	0 (0)
Exercise/Walking	4 (6.7)	5 (8.3)	0 (0)	0 (0)
Braces/support hose/kinesio tape	1 (1.7)	1 (1.7)	1 (3.0)	0 (0)
Stretching/Yoga/Tai Chi	4 (6.7)	2 (3.3)	0 (0)	0 (0)
TENS unit	0 (0)	1 (1.7)	0 (0)	0 (0)
Acupuncture	0 (0)	1 (1.7)	0 (0)	0 (0)
Meditation	0 (0)	1 (1.7)	0 (0)	0 (0)
Rest	1 (1.7)	0 (0)	0 (0)	0 (0)
Paraffin bath	0 (0)	0 (0)	0 (0)	1 (3.0)
Total # of NPMs, *Median (IQR)*	2 (2)	2 (2)	0 (1)	0 (1)

* *Denotes missing data. TENS, transcutaneous electrical nerve stimulation; IQR, interquartile range*.

### Longitudinal Changes in Brain-PAD by Pain/NPM Group

**Figure 1:**
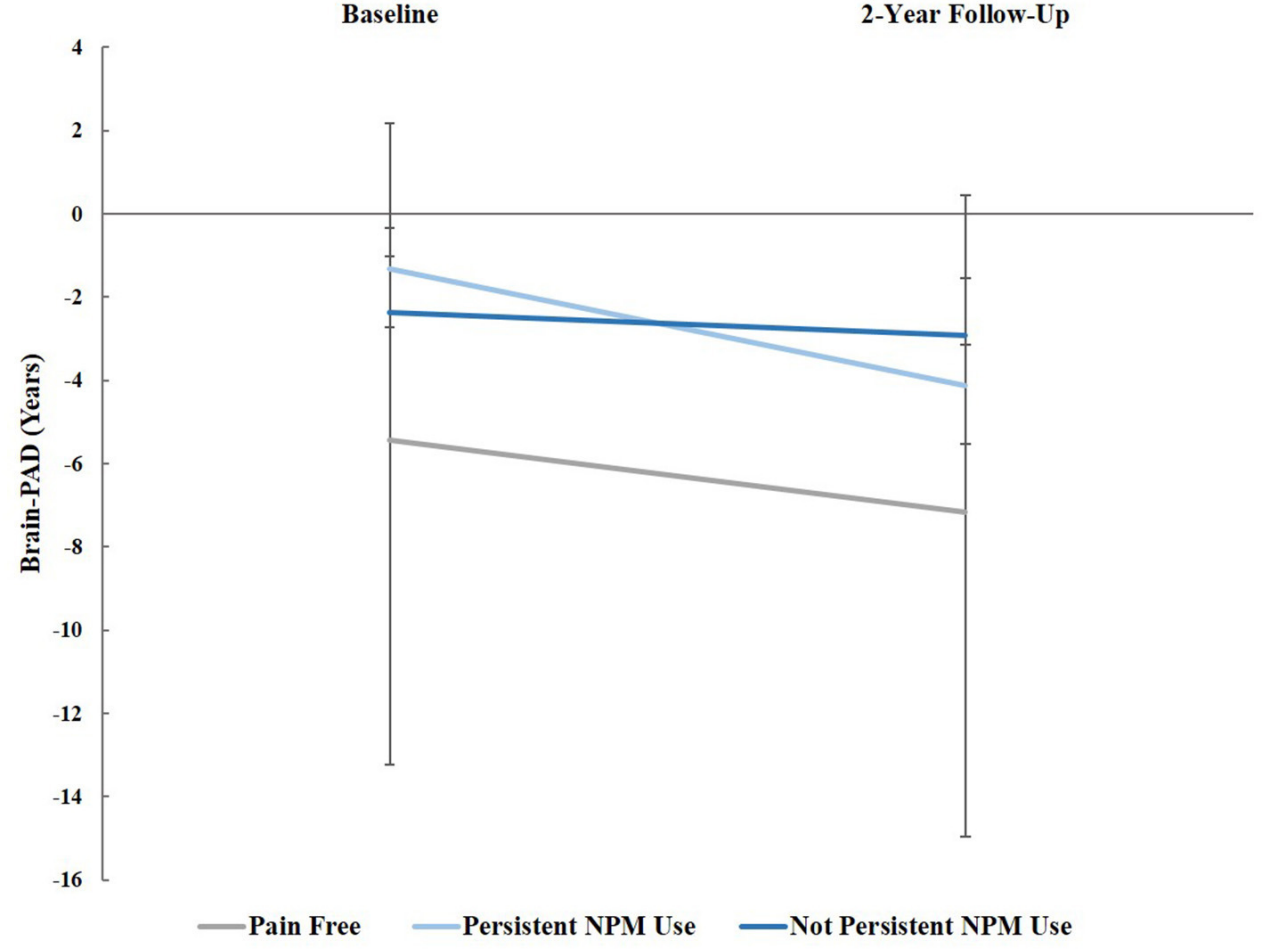
Brain-PAD at baseline and 2-year follow-up across groups, controlling for age, sex, study site, and pain grade at both time points: Pain-free, those with chronic pain using non-pharmacological pain management (NPM), and chronic pain not using NPM. Error bars represent standard error.

Repeated measures analysis of covariance (RM-ANCOVA) was used to compare brain-PAD at baseline and two-year follow-up between pain/NPM groups, controlling for age, sex, study site, and pain duration at two-year follow-up. There was a significant time (baseline and two-year follow-up) by pain/NPM group (pain free, chronic pain/persistent NPM, and chronic pain/non-persistent NPM) interaction, [*F*_(2, 79)_ = 4.25, *p* = 0.018, $\eta_{p}^{2}$ = 0.097; Greenhouse-Geisser correction], see Figure 1. Tests of simple main effects indicated that brain-PAD significantly decreased from baseline to 2-year follow-up for those with chronic pain persistently using NPM (*mean difference* = −2.803, *Bonferroni corrected p* < 0.001).

### Longitudinal Changes in Pain and Pain-Related Disability by NPM Group

**Figure 2:**
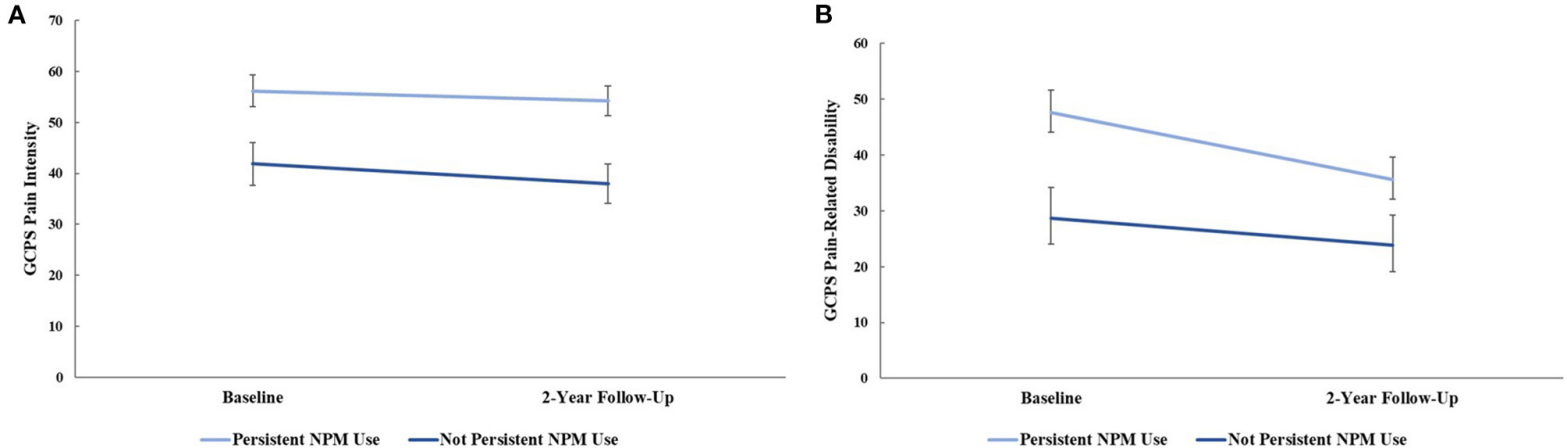
**(A)** graded chronic pain scale (GCPS) pain intensity and **(B)** GCPS pain-related disability at baseline and 2-year follow-up between non-pharmacological pain management (NPM) groups, controlling for age, sex, and study site. Error bars represent standard error.

RM-ANCOVA, adjusting for age, site, and gender, indicated a significant between-group effect for GCPS pain intensity, [*F*_(1, 89)_ = 11.28, *p* = 0.001, $\eta_{p}^{2}$ = 0.11], Figure 2A. Tests of simple main effects showed individuals who persistently used NPM had greater pain intensity at baseline (*mean difference* = 14.29, *Bonferroni corrected p* = 0.009), and 2-year follow-up (*mean difference* = 16.25, *Bonferroni corrected p* = 0.002) than non-persistent NPM users. Within-group changes in GCPS pain intensity were not significant for either NPM group (*p*'s = 0.307-0.492). A RM-ANCOVA, adjusting for age, site, and gender indicated a significant between-group effect [*F*_(1, 89)_ = 7.47, *p* = 0.008, $\eta_{p}^{2}$ = 0.077], and for GCPS pain-related disability, Figure 2B. Tests of simple main effects indicated individuals persistently using NPM had greater pain-related disability at baseline compared to those not persistently using NPM, (*mean difference* = 18.82*, Bonferroni corrected p* = 0.008). This difference was no longer significant at 2-year follow-up (*p* = 0.054). There was a significant main effect of time in those persistently using NPM, such that pain-related disability significantly decreased from baseline to 2-year follow-up (*mean difference* = −11.92, *Bonferroni corrected p* = 0.003).

## Discussion

In the present study, we sought to examine the longitudinal association between the use of common non-pharmacological pain management (NPM) strategies (e.g., ice, heat, ointments, massage) with a marker of brain aging processes [i.e., brain-predicted age difference (brain-PAD)] in a sample of community-dwelling individuals with and without chronic knee pain. Specifically, we examined whether reports of persistent NPM use over a 2-year period was associated with changes in brain aging. There was a significant time by pain/NPM group interaction in brain-PAD between the three groups (i.e., pain-free, chronic pain and persistent NPM use, chronic pain and non-persistent NPM use), such that individuals reporting persistent NPM over the two-year study period had significantly “younger” brains compared to those with chronic pain not reporting persistent NPM use.

In the current study, NPM use was assessed using a brief self-report instrument that asked about four commonly used NPM strategies in persons with chronic knee pain (i.e., ointments, cold, heat, massage). Given the lack of specific information regarding time period for use, “dose”, and other treatment-specific information, conclusions drawn from this study must be made with caution. Future studies are needed that specifically assess NPM use within a finite time period, type of NPM strategy, and application frequency to reduce the potential for misunderstanding and recall bias and to further our understanding of the neurobiological associations of NPM use in persons with chronic pain.

Interestingly, in the current study, those reporting persistent use of NPM reported significantly greater pain and pain-related disability at baseline, yet showed a significant decline in pain-related disability over time. While greater pain symptoms in those using NPM strategies may seem somewhat counterintuitive, it is likely that those experiencing greater pain were more motivated to seek out additional pain management strategies (19). Our prior work suggests that chronic pain is negatively associated with brain aging, such that greater pain is related to an “older” brain age (6). However, in the current study, we found that despite having more pain, those persistently using NPM demonstrated decreased brain aging. While caution must be taken when interpreting these findings, it is possible that use of NPM interventions for pain may buffer the negative effects of chronic pain on the brain.

Leveraging the longitudinal design of this study, we found that individuals reporting persistent NPM use had significantly “younger” brains compared to those not reporting persistent NPM use over the study period. To our knowledge, this is the first study examining longitudinal associations of NPM use with brain aging changes in a community-dwelling sample of individuals with chronic knee pain. However, the finding is in line with our prior cross-sectional work suggesting pain relief from recent NPM use may buffer from brain aging (6). Further, a randomized-controlled study of acupuncture for chronic low back pain found that 4 weeks of a standardized intervention reversed aberrant connectivity of the Default Mode Network (DMN), and that reductions in pain were related to improved DMN connectivity (20). Unfortunately, the current study did not assess the frequency or specifics of NPM use that are likely to impact efficacy (e.g., self-applied or professionally administered massage, how many times), thus, it is not known whether the average NPM ‘dose' in the current sample was sufficient to counter pain-related brain changes, especially considering the high pain burden reported by NPM users. It is likely that neurobiological alterations *via* NPM use may occur in relation to perceived pain relief, such that those perceiving benefit from NPM strategies show greater brain changes compared to those not experiencing pain relief (6). Nonetheless, this finding may represent a potential protective effect of long-term use of NPM in chronic pain conditions that are independent of clinical pain changes over time.

### Limitations

Several limitations need to be considered when interpreting the current findings. First, NPM was limited to approaches commonly used in knee OA, and that could be self-applied. This may not be representative of other NPM use which may be professionally-administered and standardized. Also, the frequency with which participants engaged in NPM is unknown in the current sample. It is likely that both frequency and duration of NPM use could influence brain aging processes. More research is needed that controls for these factors. Similarly, the current data do not reveal the degree of pain relief experienced by the participants, if any. As with our prior work, pain-relief may be a critical factor in the brain aging associations with NPM use.

## Conclusions

Overall, we found a significant association between use of non-pharmacological pain management and changes in brain aging processes in middle-aged and older adults with chronic knee OA-related pain. Yet this relationship requires more extensive investigations in larger samples and specific NPM strategies (e.g., therapeutic massage) to determine if the brain aging biomarker is related to type of NPM, experienced pain relief, or other non-specific effects. Unfortunately, we were not able to test these associations in the current work. Given the ability of the brain age paradigm to predict accelerated biological aging and functional decline prospectively (21), future studies examining standardized NPM interventions in chronic pain conditions are urgently warranted.

## Data Availability Statement

The raw data supporting the conclusions of this article will be made available by the authors, without undue reservation.

## Ethics Statement

The studies involving human participants were reviewed and approved by University of Florida, Institutional Review Board University of Alabama, Birmingham, Institutional Review Board. The patients/participants provided their written informed consent to participate in this study.

## Author Contributions

AJ and YC-A conceived of the presented idea. AJ performed the computations. JC, RF, and YC-A verified the analytical methods and supervised the project. AJ and YC-A wrote the paper with critical feedback from JC and RF. All authors discussed the results and contributed to the interpretation of the findings and approved the final manuscript.

## Funding

This work was supported by NIH/NIA Grants R01AG059809, R01AG067757 (YC-A); R37AG033906 (RF). A portion of this work was performed in the McKnight Brain Institute at the National High Magnetic Field Laboratory's Advanced Magnetic Resonance Imaging and Spectroscopy (AMRIS) Facility, which was supported by National Science Foundation Cooperative Agreement Nos. DMR-1157490 and DMR-1644779 and the State of Florida.

## Author Disclaimer

This content is solely the responsibility of the authors and does not necessarily represent the official views of the National Institutes of Health or other funding agencies.

## Conflict of Interest

The authors declare that the research was conducted in the absence of any commercial or financial relationships that could be construed as a potential conflict of interest.

## Publisher's Note

All claims expressed in this article are solely those of the authors and do not necessarily represent those of their affiliated organizations, or those of the publisher, the editors and the reviewers. Any product that may be evaluated in this article, or claim that may be made by its manufacturer, is not guaranteed or endorsed by the publisher.
